# Fourier spatial attention guided diffusion model for optimizing exposure inconsistencies in endoscopic images

**DOI:** 10.1038/s41598-026-56743-8

**Published:** 2026-06-14

**Authors:** Yan Wang, Fa Yang, Xiaoying Pan, Yiran Pan, Linjing Zhang, Danfeng Li, Yihang Wang, Kun Yang, Peng Yang

**Affiliations:** 1 School of Computer Science & Technology, Xi’an University of Posts & Telecommunications, Xi’an, 710121 China; 2 Shaanxi Key Laboratory of Network Data Analysis and Intelligent Processing, Xi’an University of Posts & Telecommunications, Xi’an, 710121 China; 3 Department of Otolaryngology-Head and Neck Surgery, Xijing Hospital, Fourth Military Medical University, Xi’an, 710075 China; 4 Department of Health Statistics, Institute for Health Informatics, Fourth Military Medical University, Xi’an, 710032 China; 5 The Ministry of Education Key Lab of Hazard Assessment and Control in Special Operational Environment, School of Public Health, Fourth Military Medical University, Xi’an, 710032 China; 6 Xi’an International Medical Hospital, Xi’an, 710100 China; 7 Xi’an Gaoxin Hospital, Northwest University, Xi’an, 710075 China

**Keywords:** Endoscope, Inconsistent exposure, Exposure correction, Diffusion model, Attention mechanism, Computational biology and bioinformatics, Engineering, Gastroenterology, Health care, Mathematics and computing, Medical research

## Abstract

Endoscopic imaging faces challenges from complex anatomical structures, limited illumination angles, and variable environmental factors, which lead to inconsistent exposure and degrade image quality and diagnostic accuracy. To address this issue, we propose FSADiff, a Fourier spatial attention guided diffusion model that integrates global frequency modeling in the Fourier domain and spatial additive attention during the inverse diffusion process to jointly address the problem of inconsistent exposure. Specifically, Fourier transform computes global correlations in the frequency domain through element-wise multiplication, enabling effective capture of overall exposure deviations. An additive attention branch then adaptively modulates the frequency-domain results in the spatial domain to suppress local degradations. In addition, we introduce a dynamic noise embedding strategy that leverages a knowledge-aware network to incorporate temporal noise information into both the denoising network and the color corrector model, thereby improving image restoration performance. We evaluate FSADiff on public datasets, Endo4IE and Endovis17, as well as a proprietary multicenter clinical nasopharyngeal dataset. FSADiff achieved superior results, yielding a Peak Signal-to-Noise Ratio of 29.00 on Endo4IE and 33.27 on Endovis17 (all 6.5+ improvement over state-of-the-art). On the clinical nasopharyngeal dataset, FSADiff achieved a Blind / Referenceless Image Spatial Quality Evaluator score of 39.17 (7.02 improvement over state-of-the-art). Further evaluation on image subsets from three hospitals demonstrated significant improvements in both overall and individual quality metrics (*p* < 0.05).

## Introduction

Endoscopes have been widely applied in the diagnostic assessment and surgical operations of human body cavities, including the gastrointestinal, respiratory, and urinary tracts^1,2^, owing to their operational flexibility, minimally invasive characteristics, and superior visualization capabilities. By acquiring high-resolution images of pathological tissues, endoscopy provides critical information for accurate disease diagnosis and treatment. However, endoscopic imaging is often limited by insufficient lumen illumination and weak probe light sources, leading to strong intensity inhomogeneity under low-light conditions. This leads to inconsistent exposure, namely, the coexistence of local overexposure and underexposure. These imaging artifacts degrade overall image quality, reduce diagnostic accuracy, and increase the risk of false positives and missed detections^3–6^. For instance, during colonoscopy, folds in the intestinal wall and occlusion of the light source often cause localized underexposure, while regions close to the endoscope’s distal light source are prone to overexposure, leading to the loss of critical mucosal feature associated with pathological tissue. Such inconsistent illumination hampers accurate visualization and consequently impairs diagnostic precision^6–8^.

Image enhancement in low-light conditions has long been a research focus in computer vision. Early approaches for image enhancement under challenging lighting conditions primarily relied on histogram-based techniques. Histogram equalization and its variants improve contrast by redistributing pixel intensities over the dynamic range. However, these methods often lead to imbalanced intensity distributions in local regions, noise amplification in dark areas, and loss of fine anatomical details^9,10^. With the rapid development of artificial intelligence, deep learning techniques have become the mainstream approach for low-light image enhancement. Guo et al.^11^ proposed a lightweight zero-reference network that enhances images by estimating pixel values within the dynamic range using high-order curves. Jiang et al.^12^ developed EnlightenGAN, an unsupervised generative adversarial network that integrates global and local adversarial losses to produce images that are normally exposed. Cai et al.^13^ combined Retinex theory with the Transformer architecture, achieving excellent results across multiple enhancement datasets. Wang et al.^14^ performed low-light images enhancement in the spatial domain by using global Fourier frequencies to effectively restore image details. Yan et al.^15^ proposed an image enhancement method based on the HVI color space. Although existing methods have achieved remarkable performance in low-light image enhancement, most are based on the assumption of uniform illumination, and focus only on the enhancement of global dark regions. Consequently, they are no well suited to the problem of inconsistent exposure where local overexposure and underexposure coexist in low-light environments. To address this challenge, LCDPNet^16^ effectively solves the problem of non-uniform exposure in natural images by explicitly modeling exposure inhomogeneity. However, inconsistent exposure in endoscopic imaging is considerably more complex due to specular reflection, tissue deformation, and dynamic light source changes^4^. Moreover, medical images demand higher requirements for detail fidelity. These challenges underscore the need for a dedicated solution tailored to inconsistent exposure in low-light endoscopic imaging.

Denoising Diffusion Probabilistic Models (DDPM)^17^, also known as Diffusion Models, have demonstrated significant potential in low-level vision tasks, such as low-light image enhancement, super-resolution, and image dehazing. By progressively adding pixel-level Gaussian noise through a forward diffusion process and decomposing the complex, unstable generation into multiple independent, stable reverse steps via a Markov chain, diffusion models achieve high-fidelity image generation^18^, offering a novel perspective for addressing exposure correction in endoscopic images. Previous studies^19,20^ have employed diffusion models to enhance illumination in endoscopic images; however, several challenges remain, retaining the standard DDPM framework leads to high computational costs, while simplifying the diffusion strategy results in performance degradation. This trade-off between efficiency and performance limits the clinical applicability of these methods^21^.

The Fourier Transform is a fundamental mathematical tool that converts signals from the spatial to the frequency domain and is widely used in image analysis and enhancement^22^. By representing an image in the frequency domain, noise can be effectively suppressed through low-frequency component processing, while edge details are enhanced by emphasizing high-frequency component. The subsequent application of the inverse Fourier Transform enables lossless reconstruction of the image in the spatial domain^23^, thereby reducing computational complexity^24^ and enhancing the efficiency of feature extraction. Existing methods mainly use Fourier Transform either directly for filter enhancement^14^ or as an independent branch in spatial-domain and frequency-domain processing^25,26^. However, these methods often rely primarily on global frequency-domain information while neglecting local details, or treat spatial and frequency domains as separate pathways. Consequently, they struggle to jointly model global features and local fine details effectively. These limitations hinder the the preservation of key medical features^27^, and fail to meet the high precision requirements of medical image processing^28,29^.

Therefore, this study proposes a Fourier Spatial Attention Guided Diffusion Model (FSADiff) to effectively address the issue of inconsistent exposure in endoscopic images. The main contributions of this study include: (a) The introduction of a Fourier Spatial Attention (FSA) mechanism that integrates Fourier Transform and additive attention as the core module of the denoising network decoder, effectively alleviating the combined effects of global and local degradations. (b) The proposal of a dynamic noise embedding method that uses a Knowledge-Aware Network (KAN) and embeds it within both the denoising network and color corrector model, enhancing image restoration under the guidance of noise temporal information. (c) Experimental results on three multi-center endoscopic datasets demonstrate that FSADiff outperforms existing image enhancement methods and achieves significant improvements in image quality.

## Methods

### Overall structure

**Fig. 1:**
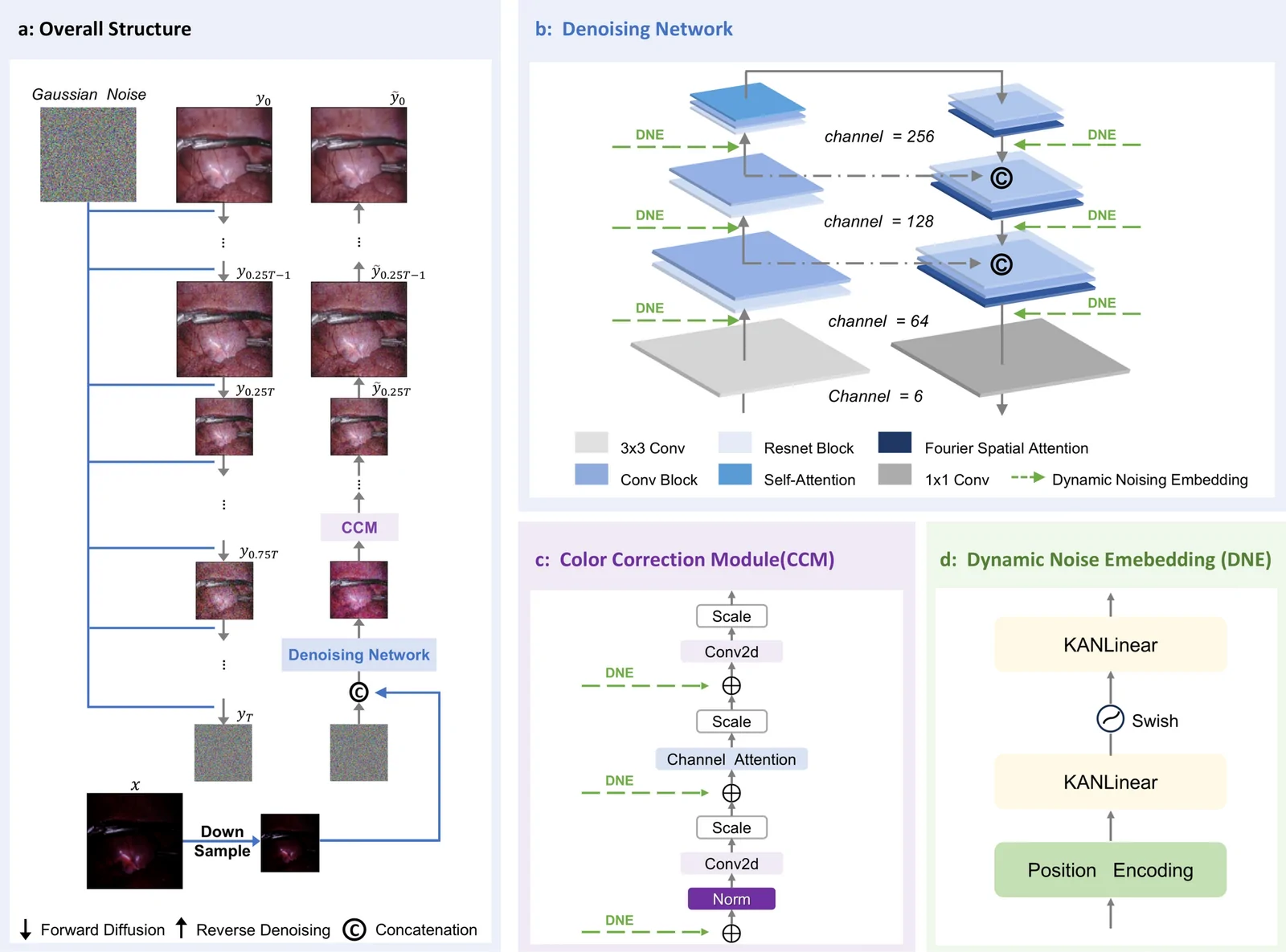
An overview of the proposed FSADiff framework. (**a**) illustrates the over architecture of the diffusion model, where image resolution is reduced at the quarter-way point of the diffusion process. (**b**) shows the detailed architecture of the denoising network, which incorporates Fourier Spatial Attention (FSA) in the decoding stage to enhance both global modeling and fine detail capture. (**c**) presents the architecture of the Color Correction Module (CCM). (**d**) shows the dynamic noise embedding mechanism, where noise features are embedded into both the denoising network and CCM to improve reconstruction accuracy.

The overall framework of the proposed FSADiff model is shown in Fig. 1. In the forward process of the diffusion model, Gaussian noise *(ζ )* is gradually added to the input image ${y}_{0}$ at full resolution within the *T/4* time steps. At the quarter point(*t=T/4*), the image is downsampled by a factor of 2 to reduce the resolution, and diffusion continues at this resolution; in the reverse process, the low-quality image *x* is used as the diffusion condition^30^. Starting from ${y}_{T}$, the denoising network processes low-resolution images during *T/4<t<T*, restores the resolution at *t=T/4*, and completes denoising at full resolution. The Color Correction Module (CCM) performs color correction on the reconstructed image at each step.

This non-constant resolution strategy aligns with LighTDiff^31^ and differs from pyramid diffusion^32^, which changes resolution at each quarter point, and can effectively reduce computational overhead. To enhance the features of key regions, a single-head self-attention is introduced in the bottleneck layer of the denoising network. FSA is used in each decoding layer to preserve detailed information. Additionally, noise information is dynamically embedded into the denoising network and CCM to provide noise temporal compensation for inverse diffusion, alleviating the degradation of model performance caused by the adjustment of the diffusion strategy. The network is optimized using the Smooth L1^33^ loss function.

### Denoising network

Due to FSADiff’s adoption of a non-constant sampling strategy^31^, the model encounters challenges in effectively balancing global and local information, resulting in a loss of fine local details in the generated images. Given the excellent performance of U-Net in hierarchical feature fusion and its concise, efficient architecture^18^, this study designs a noise-aware U-shaped denoising network to optimize the reverse denoising process. The network integrates multi-scale feature extraction, attention mechanisms, and dynamic noise embedding strategies to facilitate robust and precise image restoration across varying noise conditions. As illustrated in Fig. 1(b), its architecture consists of an encoder and a decoder, with noise temporal embedding incorporated at each network layer. The encoding stage aims to extract multi-scale features from the input image, starting with a 3×3 convolution that expands the channel dimension to 64. This is followed by multi-level ResNet and convolutional blocks, with downsampling at each stage to increase channel depth and capture features at varying scales. A single-head self-attention mechanism at the bottleneck enables global feature modeling and enhances representation.

The decoder stage restores image resolution while integrating features extracted during encoding. Mirroring the encoder’s architecture, it consists of multi-level ResNet and convolutional blocks. Feature size is progressively restored via upsampling, with multi-scale fusion achieved through skip connections. To enhance decoding performance, a Fourier Spatial Attention layer is incorporated at each decoder stage, improving feature representation. Finally, a 1×1 convolution reduces the channel dimension to three, producing the output image.

### Fourier spatial attention

**Fig. 2:**
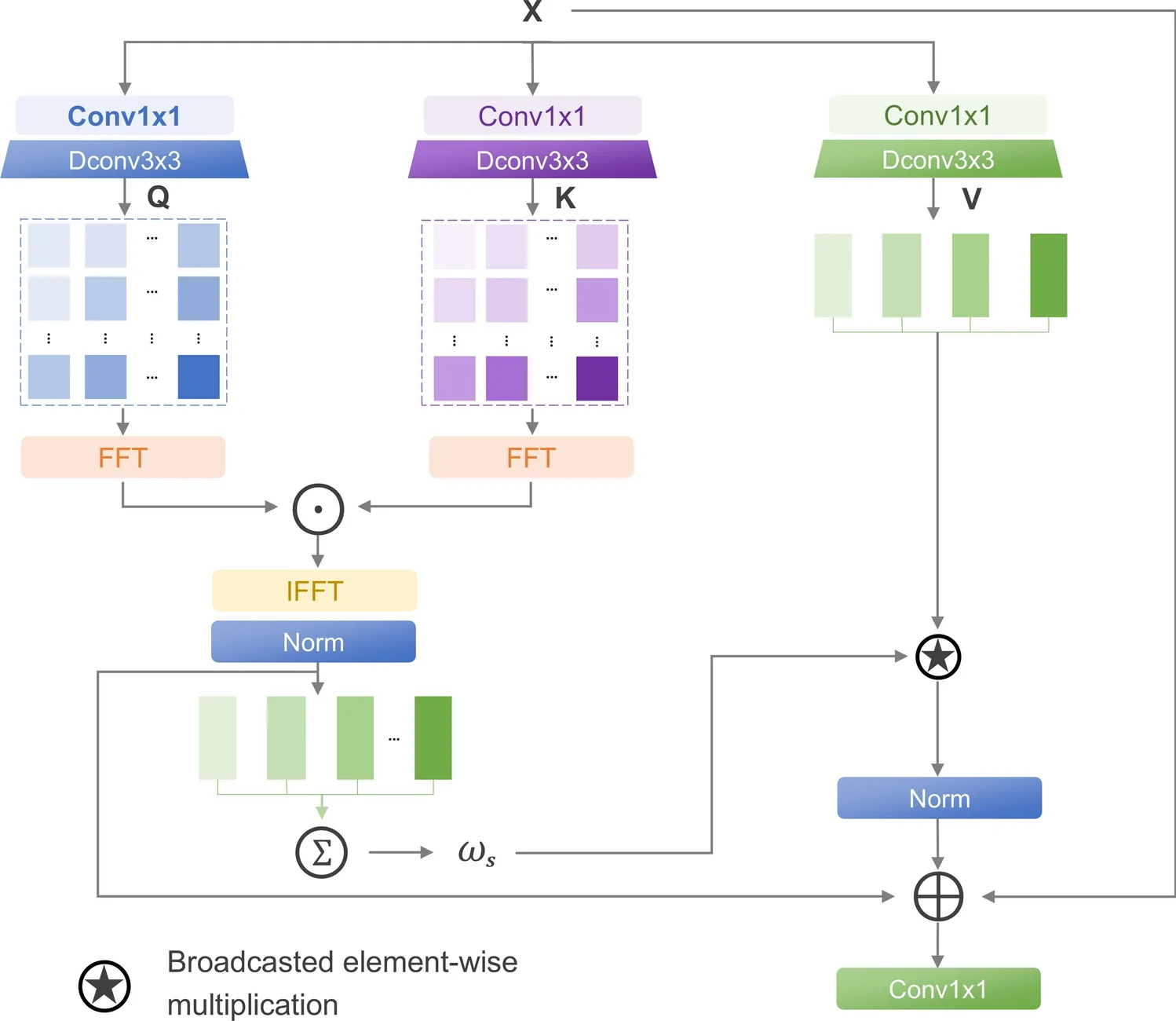
The structure of the proposed Fourier Spatial Attention. The correlation between the *Q* and *K* components of the input features is computed in the frequency domain to generate frequency-domain attention weights. These weights are then mapped back to the spatial domain via the inverse Fourier Transform, producing frequency-domain attention features. Subsequently, additive attention is applied by combining these frequency-domain features with spatial-domain attention features. Finally, a 1×1 convolution is used to output the refined image features. Notably, element-wise multiplication is used instead of the dot product typically used in self-attention.

This study proposes a Fourier Transform mixed additive spatial attention, as shown in Fig. 2. By replacing dot products with element-wise multiplication, FSA significantly improves performance while consuming minimal computational resources. FSA receives the output of the previous convolutional block as its input at each decoder layer. For the input feature *X*, it first goes through a *1× 1* convolution and a *3× 3* depthwise separable convolution (DConv) to generate Query (*Q*), Key (*K*), and Value (*V*). Then, *Q* and *K* are partitioned into *P× P* local patches, resulting in ${Q}_{p},{K}_{p}\in {R}^{B\times C\times \frac{H}{P}\times \frac{W}{P}\times P\times P}$.

The convolution theorem^34^ states that the correlation between two signals is equivalent to the element-wise multiplication of their frequency-domain representations. Inspired by this theorem, we map ${Q}_{p}$ and ${K}_{p}$ to the frequency domain using 2D Fast Fourier Transform (FFT) and calculate their frequency-domain correlation to capture global dependencies. It is important to note that correlation calculation does not involve a flipping operation but directly measures the similarity between two signals. Accordingly, the calculation of frequency-domain attention weights can be expressed as Eq (1):

1 $${\widehat{Q}}_{p}=F\left({Q}_{p}\right),{\widehat{K}}_{p}=F\left({K}_{p}\right)\\ \omega_p=Re{(}{\hat{Q}}_p\odot{\hat{K}}_p^\ast)$$

Where ${\widehat{Q}}_{p}$ and ${\widehat{K}}_{p}$ denote the frequency-domain representations of ${Q}_{p}$ and ${K}_{p}$, F(·) represents the 2D Fast Fourier Transform, ${\omega}_{p}$ is the frequency-domain attention weight, *Re*(·) denotes taking the real part of a complex number, *⊙* signifies element-wise multiplication, and ${\widehat{K}}_{p}^{*}$ is the complex conjugate of ${\widehat{K}}_{p}$.

After obtaining the frequency-domain attention weights, the Inverse Fast Fourier Transform (IFFT) is used to map the frequency-domain weights back to the spatial domain and normalize them, yielding the global attention feature ${Y}_{f}$. This process is expressed as Eq (2):

2 $${Y}_{f}=Norm({F}^{-1}({\omega}_{p}))$$

Where *Norm(· )* denotes Layer Norm normalization, and ${F}^{-1}$ represents the 2D Inverse Fourier Transform.

To further enhance local feature representation, this study initializes a learnable additive weight matrix ${\omega}_{g}$ for channel-wise weighting of the value *V*. The specific operation involves broadcasting^35^ ${\omega}_{g}$ to all spatial positions of ${Y}_{f}$ and performing summation along the channel dimension, as shown in Eq (3), to obtain the spatial attention weight ${\omega}_{s}$.

3 $${\omega}_{s}={\sum}_{c=1}^{C}({Y}_{f}\odot {\omega}_{g})\cdot {C}^{-0.5}$$

Where *C* represents the channels, and ${C}^{-0.5}$ represents the channel scaling factor.

After normalizing ${\omega}_{s}$ along the spatial dimension, it is broadcast to all channels and element-wise multiplied by the value *V*, as shown in Eq (4), to obtain the spatial attention result.

4 $${Y}_{s}={\omega}_{s}\odot V$$

Finally, the spatial attention feature ${Y}_{s}$ and the global frequency-domain attention feature ${Y}_{f}$ are summed through a residual connection, followed by a *1× 1* convolution to adjust the channel dimension, yielding the output feature. This process is expressed as Eq (5):

5 $${Y}_{out}=Con{v}_{1\times 1}({Y}_{f}+{Y}_{s})+X$$

Where $Con{v}_{1\times 1}$ represents 1×1 convolution.

### Dynamic noise embedding

The noise level, as key input information, has a complex nonlinear mapping relationship with image data and provides important temporal context for network reconstruction^36^. Although traditional Multilayer Perceptrons (MLP) possess universal approximation capability, their fixed activations function are difficult to adaptively model spatially heterogeneous degradation patterns in medical images. In endoscopic images, factors such as reflections, mucus, variations in tissue structures, and changes in camera distance lead to noise, uneven texture, and irregular illumination distribution, resulting in pronounced spatial heterogeneity. Traditional MLP, which utilize fixed activation functions and globally coupled parameters, struggle to model these complex local features effectively. This often causes gradient fluctuations, training instability, and overfitting issues. Recent studies have shown that the Kolmogorov-Arnold Network (KAN)^37^, which employs learnable B-spline basis functions, offers smooth and piecewise adaptive nonlinear modeling. This approach effectively mitigates gradient vanishing and training oscillations commonly observed in MLP, surpasses standard MLP in both accuracy and interpretability and has demonstrated particular effectiveness as a diffusion noise predictor in medical image generation tasks^38^.

Motivated by these, we propose a KAN-based Dynamic Noise Embedding (DNE) module, which is embedded into both the denoising network and color correction module to achieve cross-module dynamic collaboration of noise level information. KANLinear enhances the expressive capability of traditional linear layers through the dual expansion of basis weights and spline weights: basis weights provide global trend modeling, while spline weights capture fine-grained nonlinear changes through locally adaptive B-spline basis functions. Since the relationship between the noise level *t* and the degradation degree of endoscopic images varies with spatial position, the local adaptability of KAN can accurately model such spatial heterogeneity.

Since FSADiff processes images at multiple resolutions, the input noise level *t* is first encoded using sinusoidal position encoding via the PositionEncoding layer to introduce relative position information about *t* into the model. The position encoding representation follows the settings in PyDiff^32^. Given an input noise level (*t*) and a current feature dimension *d*, the computation of the positional encoding is expressed as Eq (6). Where $i=\mathrm{0,1}\cdots ,d-1$, the input noise level *t* is encoded into a *d*-dimensional vector *e*.

6 $${e}_{i}=\left\{\begin{array}{c}\mathit{sin}\left(\frac{t}{{10000}^\frac{2i}{d}}\right) \quad if \quad i \quad is \quad even\\ cos\left(\frac{t}{{10000}^\frac{2i}{d}}\right)\quad if\quad i \quad is\quad odd\end{array}\right.$$

Subsequently, the first KANLinear layer maps the position-encoded feature to a higher dimension. The output of KANLinear comprises two components: base weights and spline weights. For the base weight component, the SiLU activation function is first applied to the input feature to obtain an intermediate feature ${h}_{1}$, followed by a linear transformation using the base weights ${\omega}_{b}$ to generate the base weights output ${y}_{b}$. This process is expressed as Eq (7), where *σ* denotes the SiLU activation function, and ${\omega}_{b}\in {R}^{4d\times d}$.

7 $$\begin{array}{c}{h}_{1}=\sigma \left(e\right)\\ {y}_{b}={\omega}_{b}\cdot {h}_{1}\end{array}$$

For the spline weights component, the B-spline basis of the input feature *e* is first computed. The calculation is based on the input feature and a predefined set of grid points. With the number of initialized grid points as *m* and the spline order as *k*, the B-spline basis $B(e)\in {R}^{d\times (m+k)}$. The grid points are generated by uniformly partitioning the initial interval, and their support intervals are dynamically adjusted during training to adapt to the data distribution. Subsequently, by applying spline weights ${\omega}_{s}$ to the B-spline basis, the output of this component is obtained, which can be expressed as Eq (8).

The outputs of the base weights and spline weights components are summed to produce the output ${y}_{1}$ of the first KANLinear layer (Eq (9)). Subsequently, the Swish activation function is applied to the output ${y}_{1}$ of the first KANLinear layer for non-linear transformation, as expressed in Eq (10), where *ϕ* denotes the sigmoid function.

8 $${y}_{s}={\omega}_{s}\cdot B(e)$$

9 $${y}_{1}={y}_{b}+{y}_{s}$$

10 $${h}_{2}={y}_{1}\cdot \phi ({y}_{1})$$

The second KANLinear layer maps the high-dimensional feature ${h}_{2}$ back to the *d*-dimensional space. The computation process is similar to that of the first KANLinear layer, comprising both base weights and spline weights components. Its output ${y}_{2}$ represents the embedded noise level. KANLinear can more effectively capture changes in noise levels, thereby enhancing the model’s ability to encode them. By embedding this mechanism into both the denoising network and the color correction module, the model is endowed with richer prior knowledge, enabling it to better handle data under different noise levels and improving its performance and generalization.

In endoscopic images, factors such as reflections, mucus, variations in tissue structures, and changes in camera distance lead to noise, uneven texture, and irregular illumination distribution, resulting in pronounced spatial heterogeneity. Traditional Multilayer Perceptions, which utilize fixed activation functions and globally coupled parameters, struggle to model these complex local features effectively. This often causes gradient fluctuations, training instability, and overfitting issues. Recent studies have shown that the Kolmogorov-Arnold Network which employs learnable B-spline basis functions, offers smooth and piecewise adaptive nonlinear modeling. This approach effectively mitigates gradient vanishing and training oscillations commonly observed in MLP, surpasses standard MLP in both accuracy and interpretability and has demonstrated particular effectiveness as a diffusion noise predictor in medical image generation tasks.

### Color correction module

The color correction module is derived from the global corrector in PyDiff ^32^ and the chroma balancer in LighTDiff^31^. PyDiff’s global corrector achieves overall macroscopic regulation of color, while LighTDiff enhances this by incorporating a channel attention mechanism for precise color adjustment along each dimension. In this study, we innovatively embed the noise level into the chromatic balancer through Dynamic Noise Embedding (see Fig. 1(c)). As a key parameter, the noise level contains feature information of the current time step, providing additional contextual support for the color correction process. Based on the noise level, the model can dynamically adjust the correction strategy.

### Ethics declarations and compliance

This study was reviewed and approved by the local Ethics Committee (Approval No. KY-2023-079). The requirement for informed consent was waived due to the fully anonymized patient data. All procedures were conducted in accordance with the ethical principles of the Declaration of Helsinki and relevant regulations of the Fourth Military Medical University. To safeguard participant privacy and confidentiality, all personally identifiable information was anonymized, ensuring that no personal data were discolosed.

## Experiments

### Implementation details

The proposed FSADiff was implemented in PyTorch and trained on a single NVIDIA RTX 4090 GPU with 24GB memory. The model was optimized using Adam with an initial learning rate of 1×10^−4^(${\beta}_{1}$=0.9, ${\beta}_{2}$=0.999). We employed a MultiStepLR scheduler with milestones at iterations 20k, 45k, and 90k, decaying the learning rate by a factor of 0.3. The total training iteration was 100,000 with batch size 4 and gradient accumulation of 4 steps. Data augmentation included random horizontal flipping. The diffusion process used *T*=2000 timesteps with a linear noise schedule (${\beta}_{start}$=1×10^−6^, ${\beta}_{end}$=1×10^−2^ ). The training time of FSADiff is only 8 to 10 hours.

### Datasets

To evaluate the FSADiff model’s learning and generalization capabilities in image enhancement, we selected two representative datasets, Endo4IE^2^ and Endovis17^39^, for training and testing. Additionally, a proprietary real-world dataset was constructed to further validate the model. The details of these datasets are as follows:

1. Endo4IE: This public gastrointestinal endoscopy exposure correction dataset utilizes CycleGAN^40^ to process original images without exposure issues. The overexposure subset contains 1,231 image pairs, while the underexposure subset includes 985 image pairs, all sized at 512 × 512 pixels.
2. Endovis17: It is a publicly available laparoscopic dataset from the 2017 Robotic Instrument Segmentation Challenge. The original data consists of 10 robotic surgery videos, comprising 1,800 training images and 1,200 validation images, along with corresponding mask annotations. Building upon this dataset, Chen et al.^31^generated low-light images by reducing brightness and applying random gamma adjustments^41,42^, and resized the images to *256× 256* pixels for low-light enhancement tasks.
3. Real-world dataset: To further verify the model’s generalization and practicality in real clinical scenarios, we constructed a real-world clinical endoscopic image dataset for testing. Clinical nasopharyngoscope examination videos were collected from three tertiary hospitals, and frames were extracted from these videos. The detailed image screening procedure is as follows: a) Retrospective examination videos of 65 patients from each hospital were collected. After removing invalid and consecutive frames, 962, 912, and 986 images were obtained from the three hospitals, respectively. b) Three postgraduate students majoring in clinical medicine independently assessed the quality of the above images, with evaluation dimensions including exposure consistency, blurriness, and color distortion. Each criterion was scored from 1 to 5, where a higher score indicates better image quality. Samples with large scoring discrepancies (score difference > 5) were resolved through discussion or referred to a physician for arbitration. c) After excluding extremely low-quality images, only samples with total scores within the range of 5 to 10 were retained, resulting in 238, 229, and 246 images from the three hospitals, respectively. d) Two professional otolaryngologists conducted a secondary evaluation following the standard specifications of nasal and laryngeal endoscopy. Images with tissues too far from the lens or mucosa covered by secretions were excluded. After balancing the data across the three hospitals, 200 images per hospital were finally retained, totaling 600 images. The entire data screening process was conducted out under the supervision and guidance of two clinical physicians. During data preprocessing, all images were rigorously de-identified and uniformly resized to *512× 512* pixels.

### Comparison with other methods

#### Quantitative comparisons

This study evaluates the performance of the proposed FSADiff model in comparison with state-of-the-art methods developed over the past five years, including ZeroDCE^11^, EnlightenGAN^12^, LLCaps^19^, LCDPNet^16^, PyDiff^32^, EndoUIC^20^, Retinexformer^13^, LighTDiff^31^, and HVI-CIDNet^15^. For the Endo4IE and Endovis17 datasets, we employed three widely used reference-based image quality evaluation metrics: PSNR (Peak Signal-to-Noise Ratio), SSIM (Structural Similarity), and LPIPS (Learned Perceptual Image Patch Similarity) to evaluate image quality. Table 1 presents a comparative performance analysis of various methods on the Endo4IE dataset. The results indicate that FSADiff outperforms other methods in PSNR for both overexposed and underexposed images, while ranking among the top 2 in SSIM and LPIPS. These findings indicate that FSADiff effectively mitigates overexposure and underexposure issues in endoscopic images.

**Table 1:** Performance comparison by image qualities on Endo4IE dataset.

Methods	Endo4IE - overexposed			Endo4IE - underexposed		
	PSNR↑	SSIM↑	LPIPS↓	PSNR↑	SSIM↑	LPIPS↓
ZeroDCE	14.25	71.20	0.2964	15.56	69.57	0.2787
EnlightenGAN	15.66	72.90	0.2713	17.34	72.13	0.2464
LLCaps	26.49	83.37	**0.1418**	26.37	82.33	**0.1586**
LCDPNet	22.31	78.54	0.2483	23.42	79.08	0.2411
PyDiff	27.60	**87.71**	0.1956	24.28	85.03	0.2331
EndoUIC	24.94	84.00	0.2026	25.03	81.51	0.1882
Retinexformer	27.78	86.94	0.1964	28.26	85.81	0.1952
LighTDiff	28.49	86.33	0.1788	28.35	85.60	0.1788
HVI-CIDNet	22.21	82.14	0.1804	22.59	81.07	0.1892
**FSADiff(Ours)**	**29.03**	87.06	0.1761	**29.09**	**86.02**	0.1731

^*^bold indicates the best performance, and underlines denote the second-best.

Table 2 presents the comparative analysis of performance metrics and parameter counts for various methods evaluated on the Endovis17 dataset. FSADiff outperforms existing methods across both PSNR and LPIPS metrics, ranking second in SSIM. The parameter count of FSADiff is only 23.76M, ranking third among diffusion-based methods (LLCaps^19^, PyDiff^32^, EndoUIC^20^, LighTDiff^31^). Considering all three metrics collectively, FSADiff exhibits a notable balance between high performance and model complexity.

**Table 2:** Performance comparison by image qualities on Endovis17 dataset.

Methods	EndoVis17			Param(M)
	PSNR↑	SSIM↑	LPIPS↓	
ZeroDCE	11.23	31.34	0.4980	**0.079**
EnlightenGAN	24.97	83.55	0.1210	114.35
LLCaps	31.88	93.53	0.0589	119.82
LCDPNet	14.43	46.39	0.2543	0.64
PyDiff	31.02	94.50	0.0510	97.88
EndoUIC	31.73	95.26	0.0442	14.36
Retinexformer	32.69	**96.48**	**0.0362**	1.61
LighTDiff	32.89	95.45	0.0378	18.86
HVI-CIDNet	26.25	93.09	0.0502	1.88
**FSADiff(Ours)**	**33.27**	95.51	**0.0362**	23.76

^*^bold indicates the best performance, and underlines denote the second-best. “Param” represents the model parameter count.

**Fig. 3:**
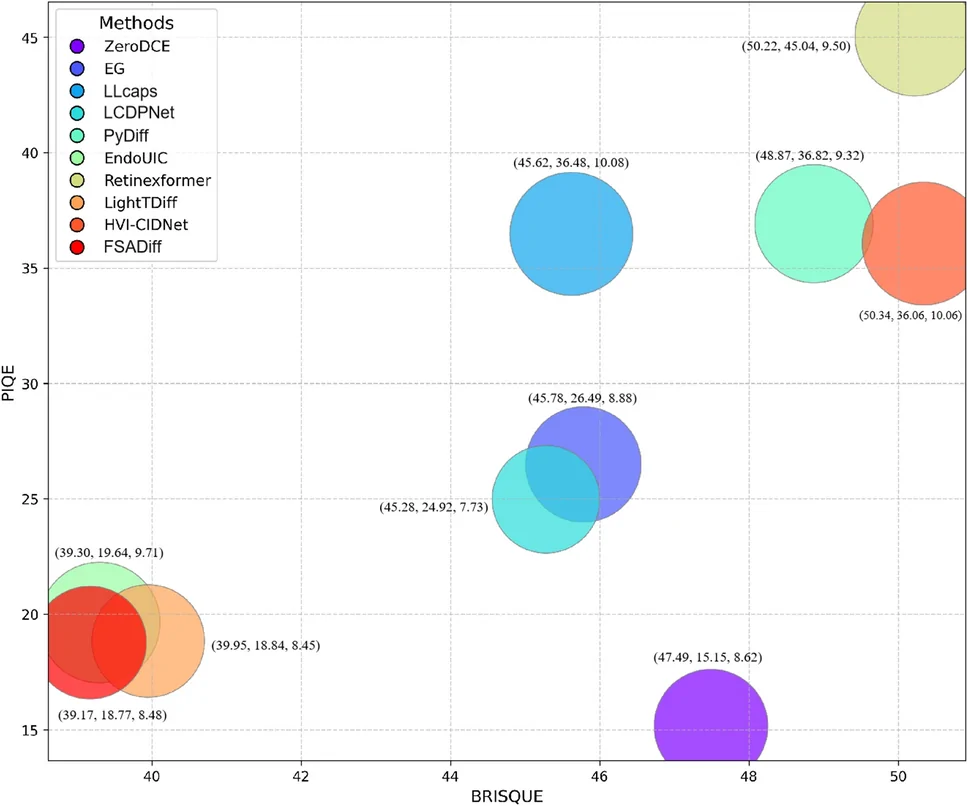
Performance comparison of image qualities on a real dataset using a bubble chart. The x-axis represents the BRISQUE metric, and the y-axis represents the PIQE metric. The size of the circles reflects the NIQE metric. Each bubble represents a method, with the three numbers displayed above the bubble corresponding sequentially to the BRISQUE, PIQE, and NIQE scores of that method. In the coordinate axes, circles locate closer to the lower-left with smaller area represent the better algorithm performance. As shown in the figure, FSADiff demonstrates the best overall performance.

For the clinical nasopharyngeal dataset, due to the lack of ground truth images, we conducted direct evaluation using three widely accepted no-reference image quality assessment metrics: BRISQUE (Blind/Referenceless Image Spatial Quality Evaluator), NIQE (Natural Image Quality Evaluator) and PIQE (Perception-based Image Quality Evaluator) to assess image quality quantitatively. The comparative results, presented in Fig. 3, demonstrate that FSADiff achieves stable and competitive performance on this real-world dataset, ranking first in BRISQUE, while also obtaining favorable results in PIQE and NIQE. These results indicate that FSADiff delivers robust image enhancement across diverse and unknown lighting conditions.

Notably, although the latest image enhancement method HVI-CIDNet demonstrates impressive performance in natural image enhancement, its effectiveness in endoscopic image processing remains limited. Specifically, on the Endo4IE dataset, FSADiff outperforms HVI-CIDNet by improving PSNR by 6.82, SSIM by 4.92, and reducing LPIPS by 0.0043 for overexposed images; for underexposed images, PSNR increases by 6.5, SSIM by 4.95, and LPIPS decreases by 0.0161. On the Endovis17 dataset, FSADiff achieves a PSNR gain of 7.02, SSIM improvement of 2.42, and LPIPS reduction of 0.014 compared to HVI-CIDNet. In the clinical nasopharyngeal dataset, FSADiff reduces BRISQUE by 11.17 and NIQE by 1.58, with PIQE values significantly lower than those of HVI-CIDNet. These results collectively demonstrate that FSADiff exhibits good applicability and consistent improvement in endoscopic image enhancement, particularly showing consistent improvement across exposure types including overexposed, underexposed, and mixed exposure scenarios.

Overall, FSADiff demonstrates best performance in PSNR and LPIPS metrics (Tables 1 and 2), occasionally ranking second in SSIM. This performance variability stems from the inherent differences in the optimization objectives of these evaluation metrics. PSNR prioritizes pixel-level fidelity, where FSADiff excels due to the precise temporal guidance provided by DNE. LPIPS measures deep perceptual similarity, and FSADiff’s frequency-space collaborative modeling effectively preserves high-level perceptual quality. In contrast, SSIM evaluates local structural similarity based on luminance, contrast, and correlation statistics within small windows. The progressive denoising mechanism in diffusion models may introduce subtle local texture variations, slightly affecting SSIM scores without compromising overall structural integrity and perceptual realism of images. Therefore, the PSNR and LPIPS performance of the FSADiff algorithm indicate that it achieves the best balance between pixel accuracy and perceptual quality.

#### Visual comparisons

**Fig. 4:**
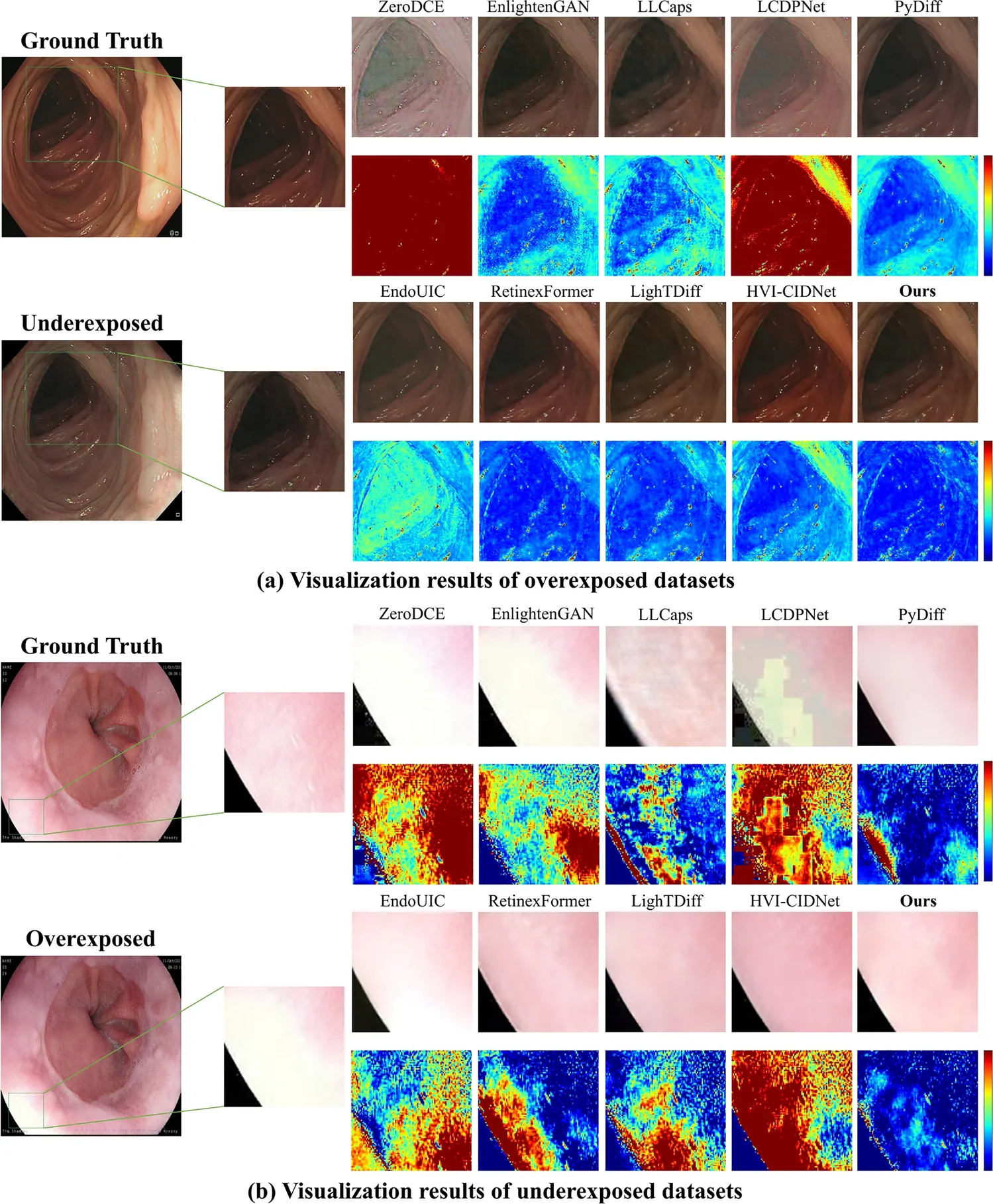
Visual comparison results of different methods on the Endo4IE dataset. Figures (**a**) and (**b**) are taken from the overexposed and underexposed datasets, respectively. The 1st and 3rd rows show the enhanced images, and the 2nd and 4th rows show the corresponding heatmaps, which illustrate the discrepancy between the processed results and the ground truth, with redder colors indicating larger errors and bluer colors indicating more minor errors. Abnormal exposure regions are magnified for detailed visualization, and the corresponding errors are quantified.

Visual analysis provides a clear, intuitive illustration of performance disparities across methods. In this study, we present enhancement results of multiple approaches on the Endo4IE dataset (Fig. 4). Regions exhibiting abnormal exposure are magnified for detailed examinations, and error heatmaps derived from the ground truth are generated to enable a quantitative comparison. For underexposed region processing, ZeroDCE and LCDPNet tend to produce blurrier images, while HVI-CIDNet enhances color but results in excessive saturation, deviating from the ground truth. In contrast, FSADiff effectively restores underexposed areas while preserving surrounding fine detail with minimal error. In overexposed regions, ZeroDCE and EnlightenGAN still exhibit extensive overexposure after processing, LLCaps mitigates overexposure but introduces artificial textures, LCDPNet produces grid-like overexposed patterns. Other methods also suffer from partial information loss within these regions. FSADiff, however, achieves optimal exposure adjustment while preserving regional textures to the greatest extent, resulting in the lowest reconstruction error.

**Fig. 5:**
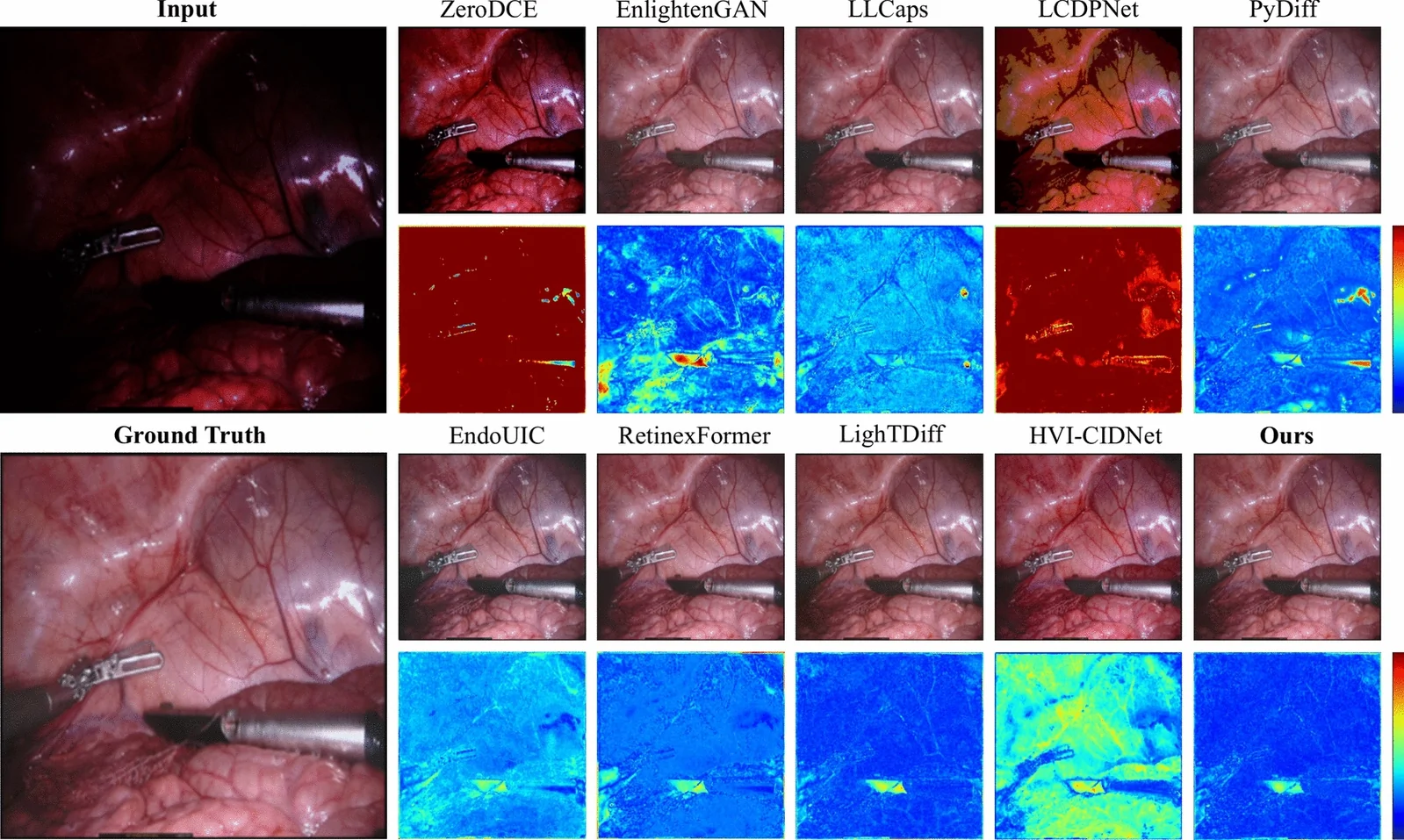
Visual comparison of different methods on the Endovis17 dataset. The 1st and 3rd rows show the enhanced images, while the 2nd and 4th rows show the corresponding heatmaps, which illustrate the discrepancy between the processed results and the ground truth, with redder colors indicating larger errors and bluer colors indicating smaller errors.

Figure 5 shows the visual comparison results of different methods on the Endovis17 dataset. ZeroDCE and LCDPNet fail to effectively enhance image illumination and have significant deviations from the ground truth, while HVI-CIDNet continues to suffer from excessive color saturation. In contrast, FSADiff achieved the lowest reconstruction error, owing to the integration of FSA and noise-time information, which significantly enhanced the model’s capability for detailed image restoration.

**Fig. 6:**
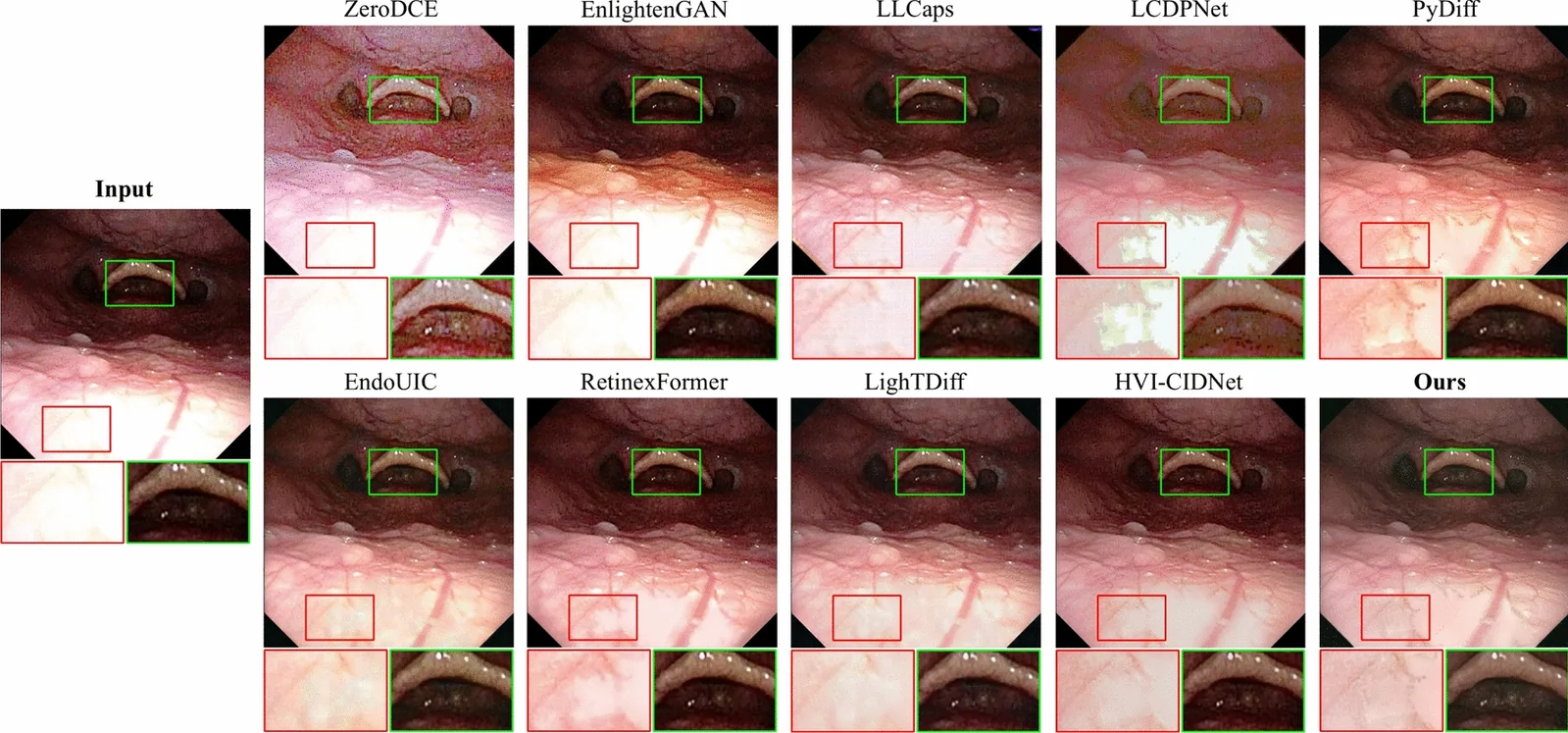
Visual comparison of different methods on real dataset. The red and green rectangular boxes show overexposed and underexposed regions, respectively.

Figure 6 shows the visual comparison of different methods on the real dataset, with magnified views highlighting regions affected by exposure issues. The image enhanced by ZeroDCE suffer severe noise contamination and obvious color distortion. EnlightenGAN also exhibits color deviation. LLCaps introduces grid-like artifacts, while LCDPNet highlights overexposed regions. PyDiff and RetinexFormer tend to over-smooth these areas (red boxed regions). EndoUIC and LighTDiff exhibit local blurring, and HVI-CIDNet suffers from a loss of fine-structure details. In contrast, FSADiff effectively preserves local details and structural integrity, thereby demonstrating superior enhancement performance.

#### Downstream segment task

**Fig. 7:**
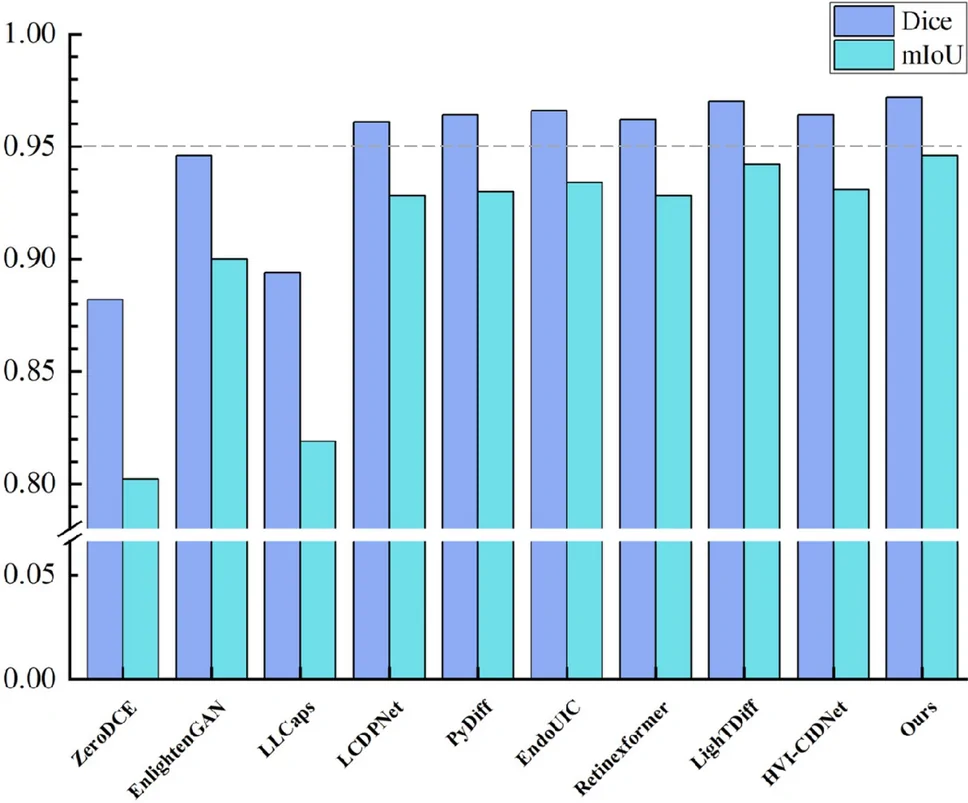
Performance comparison of downstream segmentation tasks on Endovis17 dataset. The x-axis represents different methods *(ZeroDCE, EnlightenGAN, LLCaps, LCDPNet, PyDiff, EndoUIC, Retinexformer, LightDiff, HVI-CIDNet and FSADiff)*. The y-axis represents values of the Dice metric and the mIoU metric. As shown in the figure, FSADiff achieves the best performance across both Dice and mIoU metrics.

To further validate the performance and practical applicability of FSADiff, we conducted surgical instrument segmentation experiments using CAFE-Net^43^. Specifically, CAFE-Net was first trained on the Endovis17-Seg training set, and then the pre-trained weights were used to directly evaluate the outputs of each enhancement method. As shown in Fig. 7, FSADiff outperforms other comparative methods on the Endovis17 Segmentation dataset, achieving superior Dice and mIoU scores. Compared to the suboptimal method (LighTDiff), the Dice and mIoU scores have increased by 0.2% and 0.4% respectively. This improvement in segmentation performance is attributed to the explicit handling of exposure-induced boundary degradation in endoscopic images by FSADiff. The FSA maintains the structural consistency of instrument contours by computing global frequency-domain correlation and correcting local degradation at boundaries through additive spatial attention. The CCM is responsible for eliminating color distortion that may be misjudged as boundaries by the segmentation network, while the DNE provides temporal information, further synergizing with FSA and CCM to achieve optimization. Together, these complementary mechanisms improve edge fidelity, which is critical for accurate instrument segmentation. These results demonstrate that FSADiff’s image enhancement and edge preservation capabilities provide robust data support for downstream tasks, underscoring its potential value in intelligent computer-assisted diagnosis and treatment.

### Ablation study

To validate the effectiveness of the key component in the FSADiff, we conducted ablation experiments on the Endovis17 dataset. The contributions of FSA, CCM, DNE on model performance were evaluated by systematically removing or adding each component. As shown in Table 3, in the first row, only the basic U-Net (containing only residual blocks and convolutional blocks) was used as the denoising network in the diffusion reverse process. The second row added the FSA to the basic U-Net; the third row incorporated the DNE based on the second row; the fourth row used the DNE to optimize the denoising network combined with the FSA; In the fifth row, we apply CCM without DNE to perform color correction on the output of the denoising network. Finally, the sixth row embeds the DNE within the CCM, constituting the complete FSADiff model.

**Table 3:** Ablation study on endovis17 dataset.

Denoising network		Color corrcetion module		PSNR↑	SSIM↑	LPIPS↓	Param (M)
+FSA	+DNE	-DNE	+DNE				
				29.32	94.03	0.0490	18.86
√				30.65	94.32	0.0468	19.85
	√			31.14	94.87	0.0445	22.24
√	√			31.56	94.85	0.0403	23.31
√	√	√		32.98	95.39	0.0381	23.39
√	√		√	33.27	95.51	0.0362	23.76

The results demonstrate that integrating FSA into the baseline denoising network improves performance across all evaluation metrics, indicating that FSA can accurately capture the details of endoscopic images through cross-domain feature enhancement in both the frequency and spatial domains. Adding DNE to the basic denoising network further improves the performance of the denoising network. The collaborative optimization of FSA and DNE maximizes the denoising network’s performance, demonstrating that both modules significantly enhance the accuracy of reverse denoising. The CCM effectively rectifies color deviations in endoscopic images, and when DNE is integrated into CCM to form the complete FSADiff framework, all performance indicators reach optimal levels.

### Effect on image quality improvement across three hospitals

We randomly selected 50, 100, 150, and 200 images from each of three hospitals (ED1, ED2, and ED3) to evaluate the FSADiff model’s effectiveness in improving image quality. Improvements were assessed using both combined and individual metrics across these sample sizes. For the combined analysis, BRISQUE, NIQE, and PIQE scores were jointly evaluated using Hotelling’s *T*^*2*^ test to statistically assess differences in improvements. For individual metrics, given the non-normal data distribution, the Mann-Whitney U tests were applied separately to BRISQUE, NIQE, and PIQE to compare pre- and post-enhancement results. As shown by the *Improvement Difference* in Table 4, FSADiff demonstrates a generally favorable impact on image quality metrics. Regardless of sample size 50, 100, 150, or 200, significant statistical improvements were observed across the three metrics—BRISQUE, NIQE, and PIQE (Hotelling’s *T*^2^ test, *P* < 0.05). When analyzed individually, almost all three hospitals exhibited significant improvements in BRISQUE, NIQE, and PIQE scores (*P* < 0.05). Furthermore, we observed that FSADiff yielded greater improvements on images with poorer initial quality (as indicated by higher metric values before enhancement), whereas the enhancement was less pronounced for images of moderate or good initial quality (lower metric values). For example, in the sample size 50 group, ED2 had the highest initial BRISQUE score (49.31 ± 2.9) and correspondingly exhibited the greatest improvement (10.72 ± 3.99) in the *Improvement Difference* metric (Mann-Whitney U test, *P* < 0.05). Similarly, in the sample size 100 group, ED1 had the lowest initial PIQE score (24.99 ± 8.88) and showed the smallest improvement (3.67 ± 8.25), although the enhancement was statistically significant (Mann-Whitney U test, *P* < 0.05).

**Table 4:** Effect of FSADiff on image quality improvement. random sampling of 50, 100, and 150 cases from three hospitals.

Sample size	Hospital	Before improvement ($\overline{X }\pm S$)			Improvement difference ($\overline{X }\pm S$)		
		BRISQUE	NIQE	PIQE	BRISQUE	NIQE	PIQE
50	ED1	46.79±5.01	9.07±1.23	24.85±8.82	8.13±4.33	0.45±0.99	4.14±8.07
	ED2	49.31±2.90	9.94±1.30	29.31±13.84	10.72±3.99	1.33±0.92	13.84±14.85
	ED3	46.35±4.64	8.30±0.95	30.17±5.72	6.76±4.80	0.09±0.93^**a**^	10.9±7.72
100	ED1	45.56±5.77	8.85±1.22	24.99±8.88	6.97±4.19	0.37±1.05	3.67±8.25
	ED2	49.59±3.82	9.72±1.22	30.6±13.60	10.97±4.13	1.15±0.86	14.79±14.9
	ED3	46.23±3.82	8.31±0.77	30.06±6.35	5.92±4.83	0.02±0.89^**b**^	11.03±7.82
150	ED1	45.85±5.67	8.83±1.13	25.8±9.32	7.43±3.97	0.29±0.94	4.61±8.47
	ED2	49.65±3.79	9.71±1.23	31.43±13.22	10.99±3.68	1.18±0.98	15.75±14.76
	ED3	45.47±4.33	8.30±0.81	28.28±6.77	4.61±5.01	0.02±0.81^**c**^	8.96±7.73
200	ED1	45.78±5.34	8.82±1.19	25.65±9.23	7.32±4.10	0.30±0.94	4.53±8.79
	ED2	49.47±3.76	9.84±1.32	28.81±13.42	11.21±3.78	1.21±0.99	12.89±14.73
	ED3	46.03±4.01	8.31±0.73	29.19±6.44	5.22±5.01	0.01±0.78^**d**^	9.91±7.97

a: *P*=0.49; b: *P*=0.84; c:*P*= 0.77; d: *P*=0.80.

## Discussion

This study proposes FSADiff, a model designed to address the clinical challenge of inconsistent exposure in endoscopic images. Unlike previous studies that regard low-light enhancement as a global problem, FSADiff specifically handles spatially varying over-exposure/under-exposure issues through joint frequency-domain and spatial-domain modeling. The proposed FSA mechanism combines Fourier Transform with additive attention, reducing the complexity from O(N^2^) to O(NlogN) through frequency-domain element-wise multiplication, and efficiently capturing global dependencies and local feature correlations in images. During the feature extraction process, the model can enhance the consistency of global structure while preserving edge and texture details.

To reduce the computational cost of diffusion while preserving model performance, FSADiff introduces a noise-aware U-shaped denoising network into non-constant diffusion. The dynamic noise embedding method leverages the complex nonlinear relationship between noise levels and input data to improve the generation quality of the inverse denoising process. Through elaborate experimental validation, FSADiff has shown effective improvements in various exposure scenarios and demonstrated promising potential for downstream segmentation tasks.

Experimental results demonstrate the superiority of FSADiff both quantitatively and qualitatively. FSADiff outperforms existing state-of-the-art methods in PSNR, LPIPS, and BRISQUE metrics (Tables 1 and 2). Additionally, ZeroDCE and EnlightenGAN still exhibit extensive overexposure after processing (Fig. 4(b)). Built on the diffusion model, FSADiff reconstructs images through progressive denoising and gradual restoration, enabling it to accurately characterize the complex and non-uniform illumination variations induced by high-glare, and recover textures and details in overexposed regions. By contrast, Zero-DCE relies solely on lightweight brightness curve adjustments, making it difficult to restore valid information in high-glare scenarios. Meanwhile, EnlightenGAN adopts an adversarial generation mechanism, which tends to be unstable under high-glare scenarios, typically producing oversaturated colors, exacerbated halos, blurred details, and visually unnatural outputs.

FSADiff exhibits strong generalization capabilities, consistently achieving significant improvements in image quality metrics (BRISQUE, NIQE, PIQE) across varying sample sizes (50, 100, 150, 200) (Hotelling’s *T*^*2*^ test, *P* < 0.05). These enhancements are also statistically significant in nearly all independent evaluations across the three participating hospitals (*P* < 0.05). Moreover, FSADiff demonstrates robust adaptive adjustment, providing greater enhancement for images of initially poor quality while maintaining statistically significant, though more modest, improvements for images of moderate or high quality. This adaptive mechanism enables FSADiff to transcend the limitations of the conventional “one-size-fits-all” approach, effectively restoring severely degraded endoscopic views obscured by complex anatomical structures, while avoiding over-processing or introducing artifacts in already clear images, thereby ensuring diagnostic accuracy.

Some limitations should be acknowledged. First, although FSADiff demonstrates excellent enhancement performance across three endoscopic imaging scenarios: gastrointestinal endoscopy, laparoscopic imaging, and nasopharyngoscopy, there are notable differences in enhancement efficacy among these contexts. For high-frequency interference components such as mucosal secretions in nasopharyngoscopy, FSADiff naturally modulates them in the Fourier domain. For challenges like instrument occlusion in laparoscopic imaging, FSADiff adaptively preserves occluded tissue boundaries through an additional spatial attention branch. However, the interference factors encountered in endoscopic scenarios vary significantly: Gastrointestinal endoscopy involves peristaltic movements and artifacts (bubbles, mucus); laparoscopic imaging is affected by surgical smoke and instrument occlusion; nasopharyngoscopy faces narrow spaces and uneven illumination. Consequently, domain discrepancies in feature distributions and interference types exist across these scenarios, making it challenging for FSADiff to achieve a unified and universally optimal enhancement effect under complex multi-scenario conditions. Future work will focus on integrating diverse interference types to improve the model’s generalization capability across multiple scenarios and enhance robustness under complex conditions.

Second, performance variability exists among the hospitals. This variability can be attributed, in part, to differences in endoscopic equipment models—ED1 employs the OTV-S190, ED2 uses the CV170, and ED3 utilizes the CV-260SL—as well as variations in endoscope lenses, light sources, and related systems, which affect image sensor sensitivity and illumination quality. Additionally, differences in operator technique can lead to localized over- or underexposure, resulting in lower-quality images. The cross-center variability caused by these factors poses challenges to the generality of the model in multi-center clinical applications.

Finally, the limited availability of paired images showing normal and abnormal exposure of the same anatomical site under consistent conditions constrains the feasibility of supervised model training, posing an additional obstacle to the advancement of robust image enhancement algorithms. To address these challenges, future research should focus on Masked Image Modeling^44^ and Deep Image Prior^45^. By constructing a self-supervised reconstruction framework, the model can be trained to predict missing content, thereby encouraging it to learn inherent image structures and illumination patterns. Furthermore, we plan to implement a multi-task learning framework that leverages feature sharing and the complementary nature of related tasks to simultaneously address blur and exposure for more robust endoscopic imaging. Together, these strategies could enhance the robustness and generality of FSADiff in real clinical environments, promoting its transformation from experimental research to a clinically practical tool.

## Conclusion

Endoscopic images often exhibit inconsistent exposure due to the complex anatomical structures of the examined cavities and inherent limitations in illumination angles. This study proposes the FSADiff, a novel model that effectively addresses exposure inconsistencies and achieves illumination uniformity by integrating Feature Self-Attention, Cross-Channel Modulation, and Dynamic Noise Estimation within a diffusion model framework. FSADiff demonstrates superior performance compared to existing methods on both proprietary and public endoscopic datasets. Moreover, its low-cost, non-constant resolution diffusion design enables the potential for real-time integration into clinical endoscopic systems.

## Data Availability

The datasets generated and/or analyzed during the current study are not publicly available due to patient privacy and institutional data protection policies but are available from the corresponding author on reasonable request.
